# Current and Emerging Biomarkers of Cell Death in Human Disease

**DOI:** 10.1155/2014/690103

**Published:** 2014-05-18

**Authors:** Kongning Li, Deng Wu, Xi Chen, Ting Zhang, Lu Zhang, Ying Yi, Zhengqiang Miao, Nana Jin, Xiaoman Bi, Hongwei Wang, Jianzhen Xu, Dong Wang

**Affiliations:** ^1^College of Bioinformatics Science and Technology, Harbin Medical University, Harbin 150081, China; ^2^College of Bioengineering, Henan University of Technology, Zhengzhou 450001, China

## Abstract

Cell death is a critical biological process, serving many important functions within multicellular organisms. Aberrations in cell death can contribute to the pathology of human diseases. Significant progress made in the research area enormously speeds up our understanding of the biochemical and molecular mechanisms of cell death. According to the distinct morphological and biochemical characteristics, cell death can be triggered by extrinsic or intrinsic apoptosis, regulated necrosis, autophagic cell death, and mitotic catastrophe. Nevertheless, the realization that all of these efforts seek to pursue an effective treatment and cure for the disease has spurred a significant interest in the development of promising biomarkers of cell death to early diagnose disease and accurately predict disease progression and outcome. In this review, we summarize recent knowledge about cell death, survey current and emerging biomarkers of cell death, and discuss the relationship with human diseases.

## 1. Introduction

Cell death is a fundamental biological process which has been mediated via intracellular program of biological systems [1–3]. Growing evidence has provided an expanding view for the existence of various types of cell death. Nonetheless, with different criteria, cell death can be classified into different subroutines and different subroutines of cell death own a distinct molecular mechanism and morphological characters and perform different roles in regulating the fate of cells [4]. And then along with progress and substantial insights into the biochemical and molecular mechanism exploration of cell death, its classification from the initial morphology has now been transformed to the biochemical characteristics. A functional classification suggested by the Nomenclature Committee on Cell Death (NCCD) based on the biochemical characteristics, including extrinsic as well as intrinsic apoptosis, regulated necrosis, autophagic cell death, and mitotic catastrophe, has been widely accepted [5].

In natural state cell death plays an important role during the development, maintenance of tissue homeostasis, and elimination of damaged cells [1]. One of the typical examples is that once a cell infected by virus has DNA damage or cell cycle disturbed, cell death will eliminate this cell to ensure the normal life activities of organism [1, 6]. On the contrary, excessive or defective cell death contributes to a broad spectrum of human pathologies; low-rate cell death can result in cancer formation and autoimmune disease [7–9], while high-rate cell death can result in neurodegenerative disease, immunodeficiency, and muscle atrophy [10–13]. Insights into the molecular mechanisms involved in cell death will likely have important implications and offer the opportunity to target this process for therapeutic purposes. However, the rational treatment design and selection are often precluded due to the lack of adequate biomarkers for stratifying patient subgroups. Therefore, central to current research and clinical efforts is the need for finding cell death biomarkers for early detection, diagnosis, and prognosis that can provide more accurate personalized management [14]. In this review, we summarize recent literatures on cell death biomarkers and discuss the relationship with human diseases.

## 2. Extrinsic as well as Intrinsic Apoptosis

**Figure 1:**
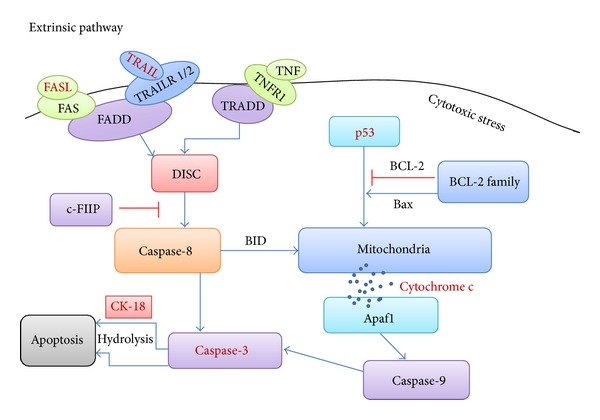
A schematic diagram of apoptosis.

Apoptosis, being a highly complex and sophisticated process, involves a series of complex biochemical events leading to a spatiotemporal sequence of morphological changes, such as nuclear condensation and fragmentation, as well as plasma membrane blebbing [15]. Characteristic biochemical events of cells undergoing apoptosis include activation of effectors caspases (caspase-3, caspase-6, and caspase-7), mitochondrial outer membrane permeabilization (MOMP), and activation of catabolic hydrolases [16]. Apoptosis can occur via extrinsic and intrinsic pathways, which are initiated either by extracellular death receptors, such as FAS, TNF-*α*, and TRAIL, or by intracellular stimuli, such as DNA damage, hypoxia, and nutrient deprivation [12] (Figure 1). Of note, the signaling cascades triggering intrinsic apoptosis are highly heterogeneous, whose triggering can proceed in a caspase-dependent or caspase-independent manner [17]. Moreover, there is accumulating evidence that cross-talk exists between extrinsic and intrinsic pathways [18].

Extrinsic apoptosis is always initiated by the activation of Fas cell surface death receptor (FAS) or TNF-related apoptosis-inducing ligand (TRAIL) which can recruit the adaptor molecules, Fas-associating protein with death domain (FADD), while it also can be stimulated by TNFR1 which can recruit TNFR1-associated death domain (TRADD). The activated FADD or TRADD leads to the formation and activation of DISC activating caspase-8. As an inhibitor, c-FIIP inactivates caspase-8 to suppress the apoptosis. The activated caspase-8 promotes the activation of caspase-3, which in turn induces the characteristics of apoptosis. Intrinsic apoptosis is triggered by cytotoxic stress resulting in the activation of p53, which promotes mitochondrial cytochrome c release into the cytosol. This process can be regulated by BCL-2 family and also triggered by BID which stimulated by extrinsic pathway. The dissociative cytochrome c binds with Apaf1 from the apoptosome to activate caspase-9. Then caspase-3 will be activated by caspase-9 finally resulting in apoptosis. In addition, caspase-3 promotes the apoptosis through hydrolyzing Ck-18 during the final stage.

## 3. Autophagic Cell Death

**Figure 2:**
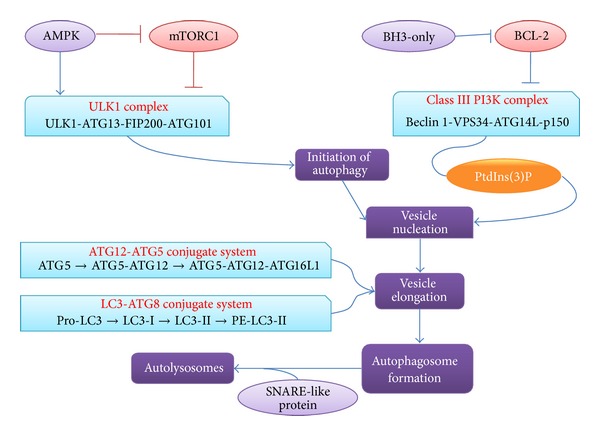
A schematic diagram of autophagic cell death.

Autophagy is an essential and conserved catabolic process, which is initiated by the nucleation of isolation membrane [19] (Figure 2). This is followed by the expansion of this membrane to form the autophagosome and fuse with the lysosome to degrade cellular components [20]. Autophagic cell death is mediated by autophagy and autophagy-related proteins and that is characterized by mTOR suppression as well as Atg activation and reaction [21]. When subjected to a variety of stress stimuli, such as energy depletion or nutrient deprivation [22], autophagic cell death can be initiated by the enhanced autophagic flux, which can also be prevented by the suppression of autophagy by chemicals and/or genetic means, such as agents targeting VPS34 or RNAi targeting essential autophagic modulators, such as ATG5 or Beclin 1 [23, 24]. It should be noted that the precise molecular mechanisms regulating autophagic cell death remain to be determined [25].

The initiation of autophagy is triggered during the starvation environment which leads to the activation of AMP-activated protein kinase (AMPK) and inactivation of the rapamycin complex 1 (mTORC1). Both of these two mechanisms can promote the formation and activation of ULK1 complex which consists of ULK1, ATG13, FIP200, and ATG101. Vesicle nucleation mainly involves the activation of autophagy-specific class III PI3 K complex to form phosphatidylinositol-3-phosphate (PtdIns(3)P). Class III PI3 K complex can be inactivated by BCL-2, while BCL-2 homology 3- (BH3-) only proteins can induce autophagy by competitively disrupting the interaction between Beclin 1 and BCL-2. Vesicle elongation process involves two ubiquitin-like conjugation systems: ATG12-ATG5 conjugate system and LC3-ATG8 conjugate system. Once the vesicle was completed, an autophagosome formed. Then the fusion between autophagosome and lysosome is mediated by several SNARE-like proteins and forms autolysosomes.

## 4. Regulated Necrosis

**Figure 3:**
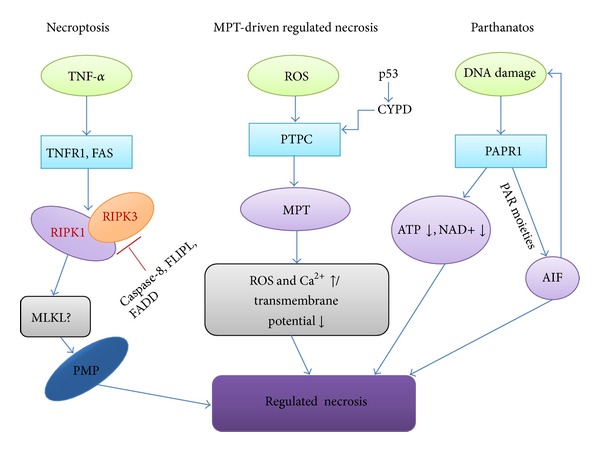
A schematic diagram of regulated necrosis.

Regulated necrosis, being a genetically controlled process, can occur in a highly regulated manner [26] and is characterized by a series of morphological changes, including cytoplasmic granulation, as well as organelle and/or cellular swelling [26]. Meanwhile, regulated necrosis is accompanied by some biochemical events, including caspase inhibition, NADPH oxidase activation, and NET release [27, 28]. Regulated necrosis can be triggered in response to a variety of physicochemical insults, including alkylating DNA damage, excitotoxins, and the ligation of death receptors [5]. Clearly, substantial advances in the characterization of the molecular mechanisms have rapidly increased our understanding of regulated necrosis. With regard to its dependence on specific signaling pathways, regulated necrosis can be further divided into different types characterized by (but not limited to) necroptosis, mitochondrial permeability transition- (MPT-) dependent regulated necrosis, and parthanatos [5, 29] (Figure 3). Of note, they are interconnected and overlapping with each other at the molecular level that impinges on common mechanisms, such as redox metabolism and bioenergetics, to result in the similar morphology [26].

Regulated necrosis at least can be divided into three different pathways, including necroptosis, MPT-dependent regular necrosis, and parthanatos. In the necroptosis pathway, the activation of TNF-alpha/FASL binds to their receptors TNFR1/FAS to activates the RIPK1 and RIPK3 which in turn phosphorylate the mixed lineage kinase domain-like (MLKL). The activated MLKL promotes the activation of plasma membrane permeabilization (PMP) and then triggers the necrosis. During this pathway, caspase-8, FLIPL, and FADD act as inhibitors of regular necrosis to suppress the activation of RIPK3. In the MPT-dependent regular necrosis pathway, transition pore complex (PTPC) plays a key role in mitochondrial permeability transition which leads to the abrupt increase of ROS and Ca^2+^ in the cytoplasm, resulting in the regular necrosis. In the parthanatos pathway, poly-ADP-ribose polymerase 1 (PARP1) starts to repair the damaged DNA, leading to the decrease of ATP and the hyperactivation of apoptosis-inducing factor (AIF) promoting the regular necrosis.

## 5. Mitotic Catastrophe

Mitotic catastrophe acts as an oncosuppressive mechanism that can occur either during or after mitosis to precede cell apoptosis, necrosis, or senescence [30]. It is characterized by unscheduled activation of cyclin B1-CDK1, TP53, or TP73, caspase-2 activation, and mitotic arrest [30–33]. Several processes have been shown to be dispensable for mitotic catastrophe that can be initiated in response to a series of triggers, including perturbation of the mitotic apparatus and chromosome segregation early in mitosis [30, 34]. During the past few years, although progress has unraveled a myriad of pathways that can induce mitotic catastrophe, it is still poorly understood [30, 35].

## 6. Current and Emerging Biomarkers of Cell Death

Currently, much attention has been given to cell death and focused on developing biomarkers, but only a few of cell death-related genes have been identified as molecular biomarkers. Most frequently described are the death receptors and their ligands, caspases, cytokeratin-18, p53, and others (Table 1). More recently, breakthroughs have identified a number of noncoding RNAs as biomarkers such as microRNAs and lncRNAs that are present in the execution of cell death [36, 37].

### 6.1. Current Biomarker

Death receptors are membrane-bound protein complexes that can activate an intracellular signaling cascade by binding specific ligands and play a central role in apoptosis [12, 38]. Death receptors belong to the TNFR (tumor necrosis factor receptor) superfamily whose members typically include Fas (also known as CD95, APO-1, and TNFRSF6), TNFR1 (also known as CD120a, p55, and p60), TRAILR1 (also known as DR4, CD261, and APO-2), and TRAILR2 (also known as DR5, KILLER, and CD262) [39]. These death receptors contain a cytoplasmic region of ~80 residues termed the death domain (DD) which provides the capacity for protein-protein interactions with other molecules [40]. Here, the most extensively studied death ligands are type II transmembrane proteins, including FasL (for Fas receptor), TNF (for TNFR1 receptor), and TRAIL (for TRAIL receptor) [41, 42]. After proteolytic cleavage of the membrane-anchored ligand, these ligands are released from the plasma membrane and enable them to bind to death receptors and trigger their activation [40]. Upon contacting with their corresponding ligands, these receptors are triggered, leading to the recruitment of a different set of adaptor molecules to the death domain and subsequent activation of the signaling cascade, where the major signals transmitted by death receptors such as Fas, TNFR1, TRAILR1, and TRAILR2 result in an apoptotic response mediated by intracellular caspases [43–48]. The above-mentioned associations give our hints and strategy guides for the death receptors and their ligands as potential biomarkers. In support of this notion, some of them have been shown to be utilized as biomarkers. For example, soluble Fas ligand is identified as a biomarker of thyroid cancer recurrence and may be useful for risk-adapted surveillance strategies in thyroid cancer patients [49]. Costagliola et al. demonstrated that TNF-alpha in tears can be used as a biomarker to assess the degree of diabetic retinopathy [50].

Caspases are a family of endoproteases that play an important role in maintaining homeostasis through regulating cell death [51]. According to their mechanism of action, caspases can be classified into two major types: one is initiator caspases, including caspase-2, caspase-8, caspase-9, and caspase-10, and the other is effector caspases, including caspase-3, caspase-6, and caspase-7. Furthermore, initiators can be subdivided into caspases that participate in either the extrinsic (caspase-8 and caspase-10) or the intrinsic (caspase-2 and caspase-9) pathway [52]. As we know, the prodomain of different caspases is different, allowing them to interact with other different molecules that regulate their activities [51]. For example, caspase-1, caspase-2, caspase-4, caspase-5, caspase-9, caspase-12, and caspase-13 contain a caspase recruitment domain (CARD), whereas caspase-8 and caspase-10 have a death effector domain (DED) [53–55]. With complexing capacity of different molecules, caspases can be activated in different ways via granzyme B, death receptors, or apoptosome [56, 57]. For example, granzyme B, which can be released by cytotoxic T lymphocytes and NK cells, is able to activate caspase-3 and caspase-7 [58]. Fas, TRAIL, TNF, and other receptors can activate caspase-8 and caspase-10 [59, 60]. Again, apoptosome that is regulated by cytochrome c and the BCL-2 family can activate caspase-9 [61]. Initiator caspases promoting the caspase cascade reaction result in the activation of effector caspases which is achieved by cleavage of their inactive proforms and then trigger the apoptotic process [51]. There has been extensive effort to identify caspase as biomarkers, the most typical example being caspase-3 [12, 62–64]. For example, Simpson et al. indicated that the active mutant caspase-3 induced by doxycycline to drive synchronous apoptosis plays key roles in human colorectal cancer cells [62]. Singh et al. speculated that caspase-3 may be a potential new biomarker for myocardial injury and cardiovascular disease [63].

Cytokeratin-18 (CK-18) and other cytokeratins constitute the type I intermediate filaments of the cytoskeleton, which is present in epithelial cells [65]. CK-18, one of the most prominent substrates for lethal caspase activation, can be cleaved by caspases, primarily not only by caspase-9, but also by caspase-3 and caspase-7, and the subsequent release of CK-18 fragments into the extracellular space occurs during cell death [66]. Notably, there are several molecular forms of CK-18 released from dying cells that can be distinguished conveniently [67]. For example, apoptosis will lead to the release of caspase-cleaved CK-18 fragments, and necrosis will lead to release of uncleaved CK-18 [68]. Multiple studies have demonstrated that CK-18 and CK-18 fragments can be released from cells into blood [66, 67, 69, 70], suggesting the potential use of CK-18 fragments or CK-18 as noninvasive biomarkers of human diseases. Vos et al. measured plasma CK-18 levels in normal weight children and obese children with and without nonalcoholic fatty liver disease (NAFLD) and found that its level is elevated in children with suspected NAFLD and was proposed as a diagnostic biomarker of NAFLD [69]. Feldstein et al. found that serum CK-18 fragment can be used as a useful biomarker for nonalcoholic steatohepatitis (NASH) in children with fatty liver disease [71]. In the review of usefulness of cytokeratin- (CK-) 18 fragments, the authors state that the caspase-cleaved fragment of cytokeratin-18 is a marker of chronic liver disease [72]. However, it should be noted that some issues with regard to the stability, reliability, and beneficial clinical utility of CK-18 and CK-18 fragments still need to be verified and answered.

In addition, DNA damage can also stimulate the transactivation of genes encoding proapoptotic proteins and trigger the apoptotic process in a p53-dependent manner [73]. The p53 is an important proapoptotic factor that is inactivated in a normal cell by its negative regulators [74]. MDM2 is the main negative regulator of p53 activity and stability [75]. A wide range of cellular stress stimuli, including DNA damage, hypoxia, and oncogene activation, can cause dissociation of the p53 from MDM2 complex [76, 77]. Once activated, p53 will induce apoptotic cell death by activating a series of positive regulators of apoptosis such as DR-5 and Bax [78, 79]. There is mounting evidence that p53 and MDM2 genes are used as biomarkers of cell death [80, 81]. Patil et al. reported using p53 as a prognostic biomarker of breast cancer [82]. Li et al. demonstrated that p53 immunohistochemical expression may serve as prognostic marker for the survival of oral squamous cell carcinoma (OSCC) patients receiving surgery [83]. Barone et al. indicated that targeting the interaction between p53 and its negative regulator MDM2 represents a new major therapeutic approach in poor prognosis of paediatric malignancies without p53 mutations [80].

### 6.2. Emerging Biomarker

Noncoding RNAs, including microRNAs and long noncoding RNAs (lncRNAs), are key regulatory molecules involved in multiple cellular processes. MicroRNAs are about 22 nt small noncoding RNA molecules, which function in transcriptional and posttranscriptional regulation of gene expression via mRNA cleavage or translational arrest [84]. It is established that they play an important role in cell death related pathway including autophagy and apoptosis [85–87]. Comparing to microRNA, lncRNAs are over 200 nt noncoding RNAs, which are emerging as new players in gene regulation as posttranscriptional regulators of splicing or as molecular decoys for microRNA [88]. Besides, some other mechanisms have been proposed to explain its mediated gene expression by lncRNA [89]. Although many lncRNAs have been identified, of all lncRNAs only few have been well characterized. Currently, emerging evidence suggests that microRNAs and lncRNAs may serve as diagnostic or prognostic biomarkers of human diseases [90]. For example, microRNA-21 (miR-21) is shown to be involved in apoptosis as well as inflammatory and fibrotic signaling pathways in acute kidney injury, which is now considered a novel biomarker aiding diagnosis and treatment of acute kidney injury [91]. MiR-497 is a potential prognostic marker in human cervical cancer and functions as a tumor suppressor by inducing caspase-3-dependent apoptosis to decrease cell growth [92]. MiR-181a functions as an oncogene by negatively regulating PRKCD, a promoter of apoptosis, to induce chemoresistance in cervical squamous cell carcinoma cells, and may provide a biomarker for predicting chemosensitivity to cisplatin in patients with cervical squamous cancer [93]. Recent demonstration that lncRNA silencing in preclinical models leads to cancer cell death and/or metastasis prevention, suggesting that they can be investigated as novel biomarkers, has triggered increasing interest [37]. An lncRNA has recently been found to play an important role in the growth and tumorigenesis of human gastric cancer and may be a potential biomarker for gastric cancer [94]. Weber et al. indicated that lncRNA MALAT1 might be applicable as a blood-based complementary biomarker for the diagnosis of non-small cell lung cancer [95].

## 7. Conclusions

In the past few years, accumulated knowledge continues to improve our understanding of the biological and biochemical processes during cell death. We have witnessed tremendous advances in the discovery and identification of novel cell death biomarkers for early detection, diagnosis, and prognosis of human diseases, with some biomarkers now in clinical utility. However, there are still debate and challenges regarding the cell death related biomarkers, including availability, stability, and accuracy of biomarkers. As far as a good biomarker is concerned, it should be present in peripheral body fluid and/or tissue such as blood, urine, and saliva. Second, it should be easy to detect, preferably in a quantifiable manner. Third, it should associate as specifically as possible with diseases. To achieve the goal, we need to find a clear path for biomarker translation from discovery to clinical practice.

## Figures and Tables

**Table 1:** Cell death biomarkers in human diseases.

Official symbol	Official full name	Clinical relevance	Function	Pathway	References
CASP3	Caspase-3, apoptosis-related cysteine peptidase	A potential new biomarker for myocardial injury and cardiovascular disease	Caspase-3 is responsible for chromatin condensation and DNA fragmentation	Apoptosis	[96]

TP53	Tumor protein p53	Implications for the regulation and execution of apoptosis in colorectal cancer and other cancers.	TP53 activation is capable of inducing apoptosis by intrinsic pathway.	Apoptosis	[97]

KRT18	Keratin 18	A biomarker of liver damage and apoptosis in chronic hepatitis C	CK18-Gly(−) involves the inactivation of Akt1 and protein kinase C*θ*	Apoptosis	[98–100]

FAS	Fas cell surface death receptor	Granulomatous disease	Fas can increase the antigen-specific CD8(+) T-cell responses during viral infection	Apoptosis	[101, 102]

TRAIL	Tumor necrosis factor (ligand) superfamily, member 10	Inducing the autoimmune inflammation in SLE	TRAIL directly induces apoptosis through an extrinsic pathway, which involes the activation of caspases.	Apoptosis	[103]

MAP1LC3A	Microtubule-associated protein 1 light chain 3 alpha	Neurodegenerative and neuromuscular diseases, tumorigenesis, and bacterial and viral infections	LC3-II functions in phagophore expansion and also in cargo recognition	Autophagy	[104, 105]

BECN1	Beclin 1, autophagy related	Human breast cancers and ovarian cancers	BECN1 is part of a Class III PI3K complex that participates in autophagosome formation, mediating the localization of other autophagy proteins.	Autophagy	[106]

RIPK1	Receptor (TNFRSF)-interacting serine-threonine kinase 1	Involving retinal disorders including retinitis pigmentosa and retinal detachment	RIPK1 and RIPK3 association forms a necrosis-inducing complex, initiates cell-death signals (programmed necrosis).	Necrosis	[107–109]

RIPK3	Receptor-interacting serine-threonine kinase 3	Atherosclerotic lesions and the pathogenesis of inflammatory bowel	RIPK3 interacts with, and phosphorylates RIPK1 and MLKL to form a necrosis-inducing complex, then triggering necrosis.	Necrosis	[110–112]
